# Preparing for the unexpected: Comparing plans for post-terror health response in Norway and France: Lisa Govasli Nilsen

**DOI:** 10.1093/eurpub/ckac130.223

**Published:** 2022-10-25

**Authors:** L Nilsen, LE Stene

**Affiliations:** 1 Section for Trauma, Catastrophes and Forced Migration - Children and Youths, Norwegian Center for Violence and Traumatic Stress, Oslo, Norway; 2 Department of Sociology and Political Science, Norwegian University of Science and Technology, Trondheim, Norway

## Abstract

**Background:**

How healthcare systems should respond to health and psychosocial needs in the population after terrorism is debated. Still, there has been recent interest for more coordinated health threat governance in Europe. Studies comparing approaches to health emergency contingency in different countries are thus needed. This poster will present a comparative study of how France and Norway planned for disaster follow-up prior to four major terrorist attacks, and how differences in the approaches can be understood.

**Methods:**

National plans and guidelines from France and Norway, planning the response to mass casualty incidents relevant to four terrorist attacks in 2011, 2015 and 2016 were analyzed, by document analysis. Walt and Gilson's health policy model, focused on context, process, content and actors guided the analysis.

**Results:**

The countries’ approaches were similar regarding identified target groups of prescribed measures and contents of some measures, however historical and systemic differences shaped approaches to post-terror needs. The countries deviated particularly on who the actors responsible for providing care were, and also the content of some measures. For instance, in France specialized mental health care were more involved in early psychosocial care than in Norway, where primary care approaches were more salient.

**Conclusions:**

Contextual factors appear to affect how healthcare contingency is planned, and finding one approach applicable in all national contexts appears challenging. Still, the presentation will discuss the potential for identifying core elements for psychosocial and healthcare follow-up that can be relevant in different contexts.

**Key messages:**

- There is variation in how countries approach the task of protecting the population’s health and psychosocial well-being following terrorism.
- There is a need to continue mapping existing practices in different countries to identify core elements for psychosocial follow-up that could be used internationally.

